# Sexually Transmitted Infections: A Novel Screening Strategy for Improving Women’s Health in Vulnerable Populations

**DOI:** 10.3390/ijms18061311

**Published:** 2017-06-20

**Authors:** Elena R. Frati, Ester Fasoli, Marianna Martinelli, Daniela Colzani, Silvia Bianchi, Luciana Carnelli, Antonella Amendola, Pierfranco Olivani, Elisabetta Tanzi

**Affiliations:** 1 Department of Biomedical Sciences for Health, University of Milan, Via C. Pascal 36, 20133 Milan, Italy; elena.frati@unimi.it (E.R.F.); ester.fasoli@unimi.it (E.F.); marianna.martinelli@unimi.it (M.M.); daniela.colzani@unimi.it (D.C.); silvia.bianchi@unimi.it (S.B.); antonella.amendola@unimi.it (A.A.); 2 Lega Italiana per la Lotta contro i Tumori (LILT), Section of Milan, Viale Molise 5, 20137 Milan, Italy; l.carnelli@legatumori.mi.it; 3 Coordinated Research Center “EpiSoMI”, University of Milan, Via C. Pascal 36, 20133 Milan, Italy; 4 CIRI-IV, Department of Health Sciences, University of Genoa, Via A. Pastore 1, 16100 Genoa, Italy; 5 NAGA Centre, Associazione Volontaria di Assistenza Socio-Sanitaria e per i Diritti di Cittadini Stranieri, Rom e Sinti, Via L. L. Zamenhof 7/A, 20136 Milan, Italy; p.olivani@libero.it

**Keywords:** undocumented migrant women, sexual health, sexually transmitted infections, prevalence, STI screening

## Abstract

Background: Migrant women are one of the most vulnerable population to health problems and well-being. This study aimed at implementing a counseling and preventive strategy for sexually transmitted infections (STIs) in undocumented migrant women in Milan, Italy. Methods: Women (ages 18–65) were enrolled at the NAGA Centre (2012–2013) and asked for a urine sample in order to carry out molecular detection of Human papillomavirus (HPV), *Chlamydia trachomatis* (*Ct*), *Trichomonas vaginalis* (*Tv*), *Neisseria gonorrhoeae* (*Ng*)-DNA. Socio-demographic and sexual behavior information were collected. All HPV/*Ct*+ women were offered Pap tests and/or were prescribed antibiotic treatment. Results: 537/757 women participated in the study (acceptability rate: 70.9%). Most of the women were from Latin America (45.6%) and Eastern Europe (30.7%); >60% of them had stable partners, did not use contraception and had had at least one pregnancy. The prevalence rates of HPV, *Ct*, *Tv* and *Ng* infections were 24.2%, 7.8%, 4.8% and 0%, respectively. In all, 43.2% of the positive women agreed to undergo a gynecological examination and accepted suitable treatment. Conclusions: This study shows an overall high prevalence of STIs in undocumented migrant women in Milan. The screening strategy based on counseling and urine testing contributed to the successfully high acceptability rate. More appropriate health services that adequately address all aspects of women’s health are required.

## 1. Introduction

We are living in the era of the greatest human mobility ever recorded in history. According to the United Nations Secretariat [1], the number of international migrants is estimated at 244 million, representing approximately 3.3% of the world population. Although they are harder to quantify, undocumented migrants are estimated to be 15–30 million worldwide (International Labor Organization, ILO) [2]. In Italy, there were 5 million migrants in 2012, and approximately 23.7% of them were living in the Lombardy Region [3].

Nearly half of all international migrants are women and girls, more and more of whom are migrating independently of the male population [4]. Therefore, migration is a factor to be considered in issues related to women, as it may lead to the worsening or improvement of women’s health. The conditions of migration, the extent of integration into the host society, the social status of the woman and the health regulations in force in the host country are all factors that affect the well-being of women. Women who migrate from low-income to high-income countries [5] are more likely to benefit from the host country’s health system, provided that they can speak the language of the host country and are employed. However, women are usually completely dependent on their husbands/partners and may not be able to access the health system to obtain adequate information and health care due to lack of health insurance and problems of male dominance [6]. In fact, migrants often move with their culture and traditional norms and this is a particular challenge for women. For example, cultural norms may prevent women from accepting care from male practitioners. Moreover, educational levels, misconceptions and prejudices hinder their access to and the success of health prevention programs. In addition, the lack of language skills can be a major barrier to understanding health care procedures and the functioning of the health system [7,8,9,10]. Therefore, in order to improve the welfare of the female migrant population, an appropriate education related to all aspects of women’s health is required, particularly in the field of reproductive and sexual health. 

Sexually transmitted infections (STIs) are one of the main public health problems affecting migrant women [11]. Most STIs are asymptomatic, but they can cause acute illnesses, chronic infections and serious delayed sequelae, such as infertility, ectopic pregnancies, cancer, long-term disability and premature deaths. Undocumented migrants are often denied access to public health services or are reluctant to use services that are available for them for fear of being deported. Even migrants who are legally entitled to health care may face various obstacles to utilizing these services [9,10]. In Italy, very few data are available regarding STI surveillance among migrant women, especially undocumented women [12,13,14]. Furthermore, although under Italian law health care is available to all migrants, including illegal migrants, no national preventive action targeted at the sexual health of female migrants has been implemented. Consequently, their access to screening is almost inexistent and the admission to medical care is limited to emergencies, when the disease has become symptomatic. In order to overcome these problems and to gain insight on sexual health in migrant women, we implemented a counseling and a preventive strategy for controlling STIs in undocumented migrant women (i.e., foreign-born person not having the official documents that are needed to enter, live in, or work in a country legally), in Milan. Urine sampling, which is non-invasive and easy to collect, was selected in order to avoid gynaecological examinations that may run counter to socio-cultural and religious beliefs, as well as to improve screening coverage.

In particular, we focused on two of the most common STIs worldwide [11], Human papillomavirus (HPV) and *Chlamydia trachomatis* (*Ct*) infections. As a secondary aim, we performed a pilot study to investigate the spread of another two STI infections, *Neisseria gonorrhoeae* (*Ng*) and *Trichomonas vaginalis* (*Tv*) in the same population.

## 2. Results

### 2.1. Study Population and Acceptability to the Study

From June 2012 to December 2013, 757 undocumented migrant women attending the NAGA Centre in Milan (Italy) [15], were invited to take part in the study, regardless of the reason (medical or otherwise) for their visit to the Centre. Informed consent, a questionnaire regarding their socio-demographic profile and sexual and reproductive health behaviour, as well as a first void urine sample were obtained from each participant. Overall, 537 women, aged 18–65 years, agreed to participate in the study. Therefore, the acceptability rate was 70.9% (95% Confidence Interval, 95% CI 67.7–74.1). Lack of time was the main reason that women (198/757; 26%) gave for refusing to participate. Twenty-two women refused to complete the questionnaire and give informed consent to the study due to the prevailing decisions of husbands/partners. This reason could have created a potential selection bias, but the proportion (22/757; 2.9%) was very low.

### 2.2. Socio-Demographic and Sexual Health Characteristics

The 537 women enrolled had a median age of 36 years (InterQuartile Range, IQR: 28–47). Subdividing for age-classes, 15.4% (*n* = 83) of women aged <25 years, 30% (*n* = 161) 25–34 years, 24.4% (*n* = 131) 35–44 years, 19.2% (*n* = 103) 45–54 years, and 11% (*n* = 59) ≥ 55 years. Regarding geographical origin, the women came from 39 different countries belonging to six World Health Organization (WHO) Regions: 45.6% (*n* = 245) of them were from Latin America [in particular, 38% from Peru, 20% from El Salvador, 17% from Ecuador, 9% from Brazil and 8% from Bolivia]; 30.7% (*n* = 165) came from Eastern Europe (47% from Romania, 22% from Ukraine, 11.5% from Albania, 9% from Moldova, 8% from Bulgaria, 2.5% from Kazakhstan, Serbia and the Russian Federation). The remaining 23.7% were from Eastern Mediterranean countries (8% (*n* = 43), for the most part from Morocco (67%)); Western Pacific (6.7% (*n* = 36), for the most part from Philippines (72%)), Africa (6.3% (*n* = 34), 32% of whom from Nigeria), and; Southeast Asia (2.6% (*n* = 14), all from Sri Lanka).

Table 1 summarizes the socio-demographic and sexual-reproductive health characteristics of the women included in the study.

A total of 46.4% of the undocumented women had been living in Italy for more than 5 years, 57.4% were married or had stable partners, and 70.4% had a high level of education (high school or degree). As for the risk factors associated with sexual health, 44.3% of women first had sexual intercourse between 16 and 19 years of age. As regards to geographical origin, women from the Eastern Mediterranean, Western Pacific, Africa and Southeast Asia first had sexual intercourse later in life (>19 years, *p* = 0.05). 65.2% of women reported to have stable sexual partners and 64.8% did not use contraceptive methods. Seventy percent of the women had had at least one pregnancy, 26.4% reported to have had any Sexually Transmitted Disease (STD) in the past. Specifically, 6.7% reported mycosis and 6.9% reported bacterial vaginosis.

### 2.3. Evaluation of DNA Quality

The β-globin gene was amplified from all 537 urine samples collected, thus confirming the suitability of the extraction protocol.

### 2.4. HPV Detection and Genotyping

Overall, the prevalence of HPV infection was 24.2% (95% CI: 20.7–28; 130/537). By subdividing for geographical origin, the percentages of HPV DNA positive women were: 29.4% (95% CI: 24.1–35.6; 72/245) for those from Latin America, 27.8% (95% CI: 15–44; 10/36) from Western Pacific, 21.4% (95% CI: 5.8–47.9; 3/14) from Southeast Asia, 21% (95% CI: 10.2–33.5; 9/43) from the Eastern Mediterranean, 18.2% (95% CI: 12.8–24.6; 30/165) from Eastern Europe, and 17.6% (95% CI: 7.5–33.1; 6/34) from Africa. Comparisons of HPV DNA positivity among the geographical groups showed a statistically significant difference for women from Latin America versus those from Eastern Europe (29.4% vs. 18.2%, *p* = 0.009).

The median age of HPV DNA positive women was 37 years (IQR: 28–47). The age-stratified HPV prevalence showed a peak among women <25 years (32.5%; 95% CI: 23.4–43.2; 27/83) while it was constantly lower in the other age groups (21.7%, 26%, 21.3%, and 20.3% in women aged 25–34, 35–44, 45–54 and ≥55 years, respectively).

No meaningful comparison can be made regarding the prevalence of HPV infection by age and geographical origin, since the women under analysis were randomly recruited (Table 2).

Overall, 77.7% (101/130) of HPV DNA positive samples were suitable (strong polymerase chain reaction (PCR) signal) for Restriction Fragment Length Polymorphism (RFLP) typing. A total of 34 different HPV types were identified from the urine samples, 18 belonging to the High Risk-clade (HR-clade: 52.9%) and 16 being Low Risk (LR) types (47.1%). Among typed infections, 85.2% (86/101) were caused by a single HPV type, while 14.8% (15/101) were multiple infections. On the whole, 67.3% (68/101) of infections were sustained by at least one type belonging to Group 1, which includes types classified as being carcinogenic for humans [16].

**Figure 1:**
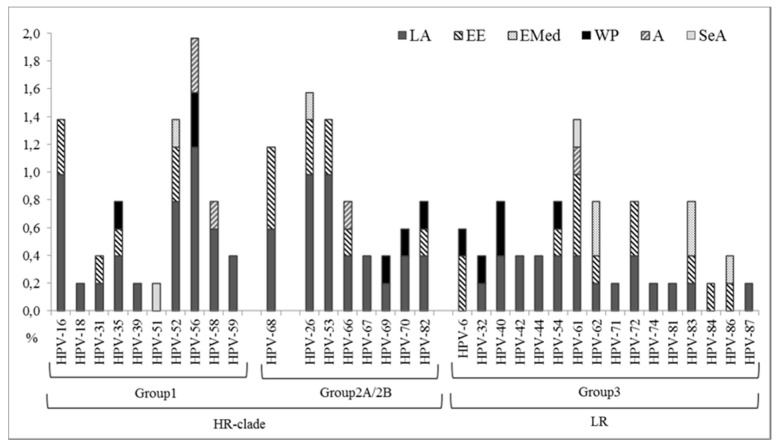
Distribution of HPV types among women (*n* = 508, excluding 29 women with untyped HPV DNA). LA, Latin America; EE, Eastern Europe, EMed, Eastern Mediterranean; WP, Western Pacific; A, Africa and SeA, Southeast Asia. According to the IARC classification system [16]: Group 1 = carcinogenic to humans; Group 2A (HPV-68) = probably carcinogenic to humans; Group 2B = possibly carcinogenic to humans; Group 3 = not classifiable as carcinogenic to humans.

Among the 508 women enrolled (excluding 29 women with untyped HPV DNA), HPV-56 was the most frequent type in Group 1, with a frequency of 2.0% (10/508), followed by HPV-16 and HPV-52 (7/508, 1.4% each). HPV-18 was only detected in 0.2% (1/508) of the women enrolled (Figure 1).

### 2.5. C. trachomatis Detection and Genotyping

Overall, the prevalence of *Ct* infection was 7.8% (95% CI: 5.8–10.3; 42/537). Among the infected women, 16.3% (95% CI: 7.4–29.6; 7/43) were from the Eastern Mediterranean region, 9.8% (95% CI: 6.5–14; 24/245) from Latin America, 8.8% (95% CI: 2.3–22.2; 3/34) from Africa, 5.6% (95% CI: 0.9–17.2; 2/36) from Western Pacific, and 3.6% (95% CI: 1.5–7.4; 6/165) from Europe. Comparisons of geographical groups showed higher percentages of *Ct* DNA positivity among women from the Eastern Mediterranean and Latin America versus European women (16.3% vs. 3.6% and 9.8% vs. 3.6%, *p* = 0.007 and *p* = 0.01, respectively).

The median age of positive women was 28.5 years (IQR: 24.2–36). The prevalence peak was observed in women <25 years (13.2%, 95% CI: 7.2–21.9) with a decrease according to age (11.2%, 4.6%, 3.9%, and 5% among women aged 25–34, 35–44, 45–54 and ≥55 years, respectively).

For the same reason mentioned above, no meaningful comparisons can be made regarding the prevalence of *Ct* infection by age and geographical origin (Table 3).

Fifty percent (21/42) of *Ct* DNA positive samples were successfully genotyped. Seven different *genovars*, belonging to *biovar* trachoma, were identified: *genovar* E was the most frequently found infecting seven samples (33.3%), followed by *genovar* G (19%, four samples), by *genovar* D and F (14.3%, three samples each), H (9.5%, two samples), Ia and Ja (4.8%, one sample each).

### 2.6. HPV/C. Trachomatis Co-Infections

Among the 537 undocumented migrant women, 17 (3.2%; 95% CI: 1.9–5) were HPV/*Ct* co-infected. There were no specific socio-demographic or sexual/reproductive health characteristics, or features of the infecting pathogen, associated with co-infection.

### 2.7. Diagnostic and Therapeutic Follow-Up

All of the 155 HPV- and/or *Ct*-infected women were contacted by phone in order to inform them of their STI status and to make a medical appointment. Overall, 28 (18.1%) women did not answer the phone despite repeated attempts, 60 (38.7%) did not attend the medical appointment for work, logistical, and/or personal reasons, and 67 (43.2%) agreed to undergo a gynaecological examination and to have suitable treatment. The outcome of the Pap test carried out on 58 HPV DNA positive women was: negative cytology in 77.6% (45/58) and atypical squamous cells of undetermined significance (ASC-us) in 32.4% (13/58). Six women (6/13; 46.2%) with ASC-us were infected with HPV types belonging to the HR-clade (HPV-16, -26, -51, -56, -66 and -82, respectively) and 7 (53.8%) were infected with LR HPV types. Nine *Ct* infected women received specific antibiotic treatment.

**Figure 2:**
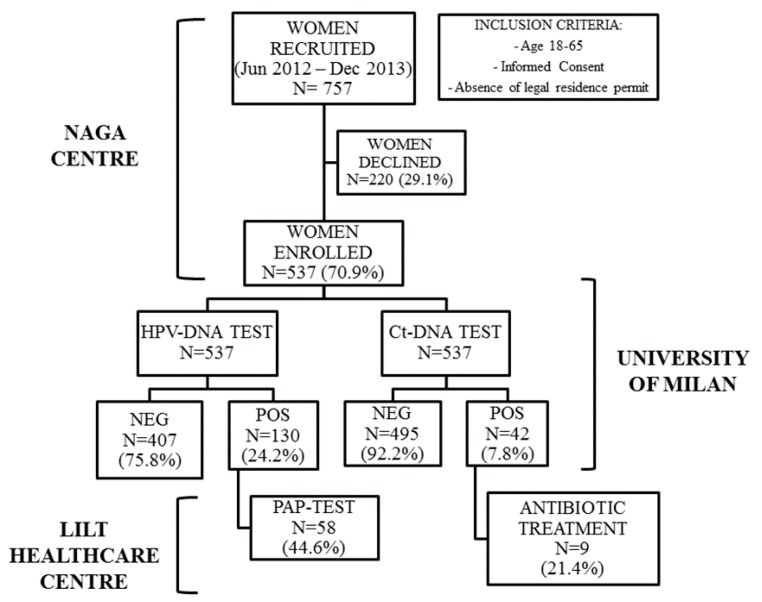
Flowchart with the study design and the main results.

The flowchart with the design of the study and the main results is reported in Figure 2.

### 2.8. N. gonorrhoeae and T. vaginalis Detection

*T. vaginalis* and *N. gonorrhoeae* DNA was detected in the anonymous residual aliquots from the 537 samples collected in the pivotal study.

The overall percentage of *Tv* DNA positivity was 4.8% (95% CI: 3.3–7.0; 26/537), with the highest infection rate (11.6%; 95% CI: 5.1–24.5; 5/43) among women from the Eastern Mediterranean region, and the lowest among those from Latin America (2.4%; 95% CI: 1.1–5.2; 26/245) (*p* = not significant). The proportion of women infected with *Tv* was 7.9% (95% CI: 4.7–13.0; 13/165) and 5.6% (95% CI: 1.5–18.1; 2/36) in Europe and in the Western Pacific, respectively. None of the 14 women from Southeast Asia were infected with *Tv*.

The median age of infected women was 39.5 years (IQR: 29.5–46.5) and the prevalence increased according to age up to 54 years (3.6%, 4.3%, 6.1%, 6.8% among women aged <25, 25–34, 35–44, 45–54 years, respectively). Seven women *Tv* DNA positive (1.3%; 95% CI: 0.6–2.7; 7/537) were also HPV and/or *Ct* co-infected: 3 women had a *Tv*/HPV co-infection, 3 a *Tv*/*Ct* co-infection and one woman had a triple infection *Tv*/HPV/*Ct*. There were no women infected with *Ng*.

## 3. Discussion

Most of the United Nations member states have ratified treaties recognizing the right to equal and equitable access to health care for all persons, notwithstanding their legal standing within a government system [8,9]. In Europe, much attention has been paid to the health of migrants due to the intensification of human mobility across continents especially in recent years. However, accurate information on migrants and their health status are not available in many European countries. In addition, both existing epidemiological and health data may have been underestimated due to difficulties in including undocumented migrants [17]. Health care access for undocumented migrants varies considerably between countries, and they are generally less likely to gain access to it than legal residents [8,9]. Not having access to appropriate health care can lead to severe outcomes that could otherwise be managed or treated. Among the migrant population, women are the most vulnerable to health problems and well-being. In this context, reproductive and sexual health and STIs in particular are the main public health issues affecting migrant women.

In some European countries, such as Austria, Denmark, Finland, Ireland, Luxembourg, and Sweden, undocumented migrants are only legally entitled to receive emergency health care, even if they pay for it, and do not have access to family planning or to regular sexual and reproductive health check-ups and screening for STIs [7,8,9,10]. Furthermore, in other countries, restrictive policies ban or impose restrictions on the over-the-counter sale of emergency contraception. For example, in Hungary, emergency contraception requires a prescription, which is often inaccessible for undocumented migrant women [7,8,9,10].

In Italy, although health care is available to all migrants under Italian law, including those who lack legal documents, no national prevention strategy is directed to female migrants, therefore they do not have access to screening programs.

The aim of this study was to outline and evaluate STI screening and to gain knowledge on the spread of HPV and *C. trachomatis* infections among undocumented migrant women living in Milan, who attend the NAGA Centre.

A large number of women participated in the study with an acceptance rate of 70.9%. This percentage of participation in molecular screening (HPV DNA testing) is much higher than that observed in previous offers of conventional cytological screening (Pap test) at the NAGA Centre (estimated acceptance rate of approximately 4%, unshown data).

Patient counseling and self-collected urine samples proved to be preferable for these women and contributed to the successful outcome of the study. During counseling women received information on the diagnosis, prevention, screening and treatment of sexually transmitted infections and diseases. This strategy has helped women to overcome their fears and prejudices related to sexual health and they were therefore more willing to undergo free screening for HPV and *C. trachomatis* infections. Urine sampling, a methodology already tested and validated by us [18,19], thus avoiding gynecological examination as well as socio-cultural and religious implications, proved to be non-invasive, easy and quick, and therefore a good alternative to conventional cervical brush for Pap screening. Furthermore, the offer of a “real time” STI screening may have been the tipping point for participation.

This was not the case for the next step of diagnosis or treatment after screening. In fact, a high loss to follow-up was observed, since only 43.2% (a low, yet significant proportion) of women positive for HPV and/or *C. trachomatis* DNA agreed to undergo further investigation. This was probably due to the long interval between the actual screening and the follow-up appointment at the medical centre, to work commitments or change of residence. LILT Health care is a voluntary medical centre with a limited availability of appointments, which was the main cause of the long period between sample collection and examination. As recommended by WHO, the screen-and-treat approach is probably the only effective preventive strategy for these women at high risk and who are hard to reach [20].

The results from this study have provided baseline data regarding the epidemiology of HPV and *C. trachomatis* infections in undocumented migrant women in Milan. The overall HPV and *C. trachomatis* prevalence were 24.2% and 7.8%, respectively, and peaks were observed in younger women, aged <25 years (32.5% and 13.2%, respectively). Nearly 70% of HPV positive women were infected by types known to be at high oncogenic risk; the most detected type was HPV-56 (2.0%), followed by HPV-16 and HPV-52 (1.4% each). These HR-HPV types are known to be the most common in women with normal cytology worldwide [21]. The prevalent *genovar* among *Ct* positive women was *genovar* E (33.3%), known to have a biological advantage over the other *genovars* thanks to its ability to escape the immune response and to have specific virulence factors, able to facilitate the transmission and infectious processes [22].

The study population was enrolled among women who freely and randomly had access to the NAGA Centre during the study period three days per week. It is therefore not representative of the geographical area of origin, especially for the small number of women from some areas compared to those from Latin American and Eastern Europe, but it reflects the migratory movement across a large urban area of Lombardy, which is the region with highest percentage of immigrants in Italy.

The prevalence of HPV and *C. trachomatis* infections detected in this cohort of irregular immigrants are not comparable to the prevalence data reported for these two STIs in both the countries of origin and the host country.

Regarding HPV infection, a meta-analysis [16] that included 194 studies on approximately one million women with normal cytological findings worldwide showed the highest HPV infection rates for women in Sub-Saharan Africa (24.0%), Eastern Europe (21.4%), and Latin America (16.1%). As observed in our study, there is a peak in the age-specific HPV distribution for young women (<25 years) [21].

The percentage of undocumented migrant women infected with HPV was higher than that reported for the Italian female population with normal cytology (HPV DNA prevalence: 15.8%) [23], but lower than the prevalence reported in other studies on migrant women in Italy (42–47.8%) [12,13]. However it is important to note that the women included in these studies came from two geographical areas at high prevalence of HPV infection (Africa and Eastern Europe), or had a history of prostitution. The discrepancies are probably due to various socio-demographic characteristics and risk factors.

The prevalence of *C. trachomatis* infection observed in this cohort of undocumented migrant women was slightly higher than that reported for Italian women (7.8% vs. 5.2%) [19]. However, this prevalence is equal to that observed in another study conducted on 233 Eastern European and Western African immigrant women in Southern Italy (7.7%) [24].

Lastly, the availability of residual DNA aliquots from urine samples collected for the main study enabled us to investigate the spread of the two other common curable STIs, *T. vaginalis* and *N. gonorrhoeae* infections, in the same population. An overall prevalence of 4.8% was observed for *Tv* infection, while no women among the undocumented migrants in Milan were infected by *Ng*. These are the first data obtained in Italy for this high-risk cohort and are comparable to the data reported in women of the general population worldwide (*Tv* prevalence: 4.0–6.4%; *Ng* prevalence: 0.6–1.0%) [25].

As regards to the migrant population, data on the prevalence of *T. vaginalis* are not available. The European prevalence data for *N. gonorrhoeae* infection are similar to that observed in the native population, but active surveillance should be continued due to the increase of this infection among migrant women in recent years (2000–2010) [25]. In addition, both infections are a major public health problem since they can cause acute inflammatory disease, premature rupture of membranes and premature birth, and increase the risk of contracting HIV [25].

Our results show an overall high prevalence of STIs in undocumented migrant women. The epidemiological profile varies according to geographical origin, since women from Latin America showed the highest rate of HPV infection while women from the Eastern Mediterranean area had the highest *Ct* and *Tv* infection rates.

## 4. Materials and Methods

### 4.1. Study Design

The study was conducted in accordance with the Declaration of Helsinki, and the protocol was reviewed and approved by the Ethics Committee of the University of Milan, Italy, in April 2012. The main study was carried out from June 2012 to December 2013 at the NAGA Centre in Milan (Italy), which is a voluntary association that provides medical and legal assistance to undocumented migrants, Rom and Sinti [15]. All undocumented female migrants who attended the NAGA Centre were invited to take part in the study by the multilingual mediators, regardless of the reason (medical or otherwise) for their visit to the Centre. Informed consent for participating in the study and for storing residual samples anonymously (by blinding all information except age and geographical origin) in a bio-bank for future analyses was obtained from all participants. Inclusion criteria of this study were: the ability to provide informed consent and communicate in Italian or English, Spanish or French, as suggested by the expert staff employed at the NAGA Centre; aged between 18 and 65 years; absence of a legal residence permit. After having given informed consent, the participants completed a self-administered questionnaire consisting of 10 multiple-choice questions regarding their socio-demographic profile (age, marital status, origin, education, migration time) and sexual and reproductive health behaviour (use of contraceptive methods, age at first intercourse, number of sexual partners, number of pregnancies, past or current STDs). The questionnaires were available in four languages (Italian, English, Spanish, and French). Researchers from the University of Milan and multilingual NAGA mediators were available on site every Monday, Wednesday and Friday mornings to help understand and fill in the questionnaire and for a private counseling on health education, diagnosis and prevention of STIs.

The women were then enrolled in the research study and asked for a urine sample for carrying out molecular analyses for STIs (HPV DNA and *Ct* DNA detection). The urine sample was selected, as it is non-invasive and easy to collect, thus potentially more attractive to women since medical examination is not required, as well as for socio-cultural and religious implications. All women found to be positive for HPV and/or *Ct* infections were informed by the investigator directly or by phone call and then examined by a gynecologist at the Lega Italiana per la Lotta contro i Tumori (LILT) Healthcare Centre in Milan [26] for further investigation or treatment, specifically a Pap test and/or antibiotic treatment, in the case of HPV or *Ct* infection, respectively. This is a voluntary medical organization.

### 4.2. Sample Collection and DNA Extraction

First void urine samples were obtained from each woman in sterile containers, which were stored at room temperature (RT) and processed within 6–8 h after collection at the Virology Laboratory of the Department of Biomedical Science for Health, University of Milan. In order to obtain the cellular component, at least 15 mL of each sample was pre-treated by means of two different successive steps of centrifugation and washing, as previously described [18] and stored at −20 °C until nucleic acid extraction. DNA was extracted from 500 μL of concentrated urine samples using the NucliSENS^®^ easyMAG™ automate (bioMérieux bv, Lyon, France) method following the standard protocol with off-board lysis. The elution volume was 100 μL. The concentration and purity of the DNA extracted were evaluated using a spectrophotometer (NanoDrop™ 2000 c, Thermo Fisher Scientific, Inc., Wilmington, Germany). We determined the quality of the DNA by detecting a 268 base pair (bp) fragment of the housekeeping beta-globin gene using an *in house* polymerase chain reaction (PCR) assay [27]. The DNA extracted was used for HPV and *Ct* molecular analyses and the residue was stored at −20 °C for further analyses.

### 4.3. Molecular Assays

#### 4.3.1. HPV Detection and Genotyping

The PCR-based method utilized in this study was previously validated using paired cervical and urine samples, showing high sensitivity and specificity for the detection and genotyping of HPV DNA in urine samples [18]. Briefly, HPV DNA was detected through PCR amplification of a 450 bp segment of the Open Reading Frame (ORF) L1 using the degenerate primer pair ELSI-f and ELSI-r. Every PCR reaction included positive (HPV-16 positive cells, Caski) and negative (water) controls. All amplified fragments were then subjected to viral type analysis by means of the RFLP method that is capable of identifying all HPV types (HR and LR types) of the alpha genus according to the latest IARC classification system (HR-clade, group 1: HPV-16, 18, 31, 33, 35, 39, 45, 51, 52, 56, 58, 59; group 2A: HPV-68; group 2B: HPV-26, 30, 34, 53, 66, 67, 69, 70, 73, 82, 85; LR types: HPV-6, 11, 28, 32, 40, 42, 43, 44, 54, 55, 57, 61, 62, 71, 72, 74, 81, 83, 84, 86, 87, 89) [16].

#### 4.3.2. *C. trachomatis* Detection and Genotyping

*Ct* DNA was detected through nested PCR amplification of a 150 bp fragment of the cryptic plasmid [28]. Each PCR run included positive (*Ct* positive urine sample) and negative (water) controls. In order to identify *Ct genovar*, all positive samples underwent sequencing following emi-nested PCR amplification of a 993 bp fragment of the *ompA* gene [28]. The sequences obtained were compared with reference strains present in the GenBank database (BLASTn) [29] to establish the *genovar*.

#### 4.3.3. *N. gonorrhea* and *T. vaginalis* Detection

Once the main study was concluded, the residual DNA was used in an *in house* duplex RealTime PCR (Applied Biosystems 7300 Real Time PCR System, Thermo Fisher Scientific, Inc., Wilmington, Germany) in order to detect the presence of *Ng* and *Tv* infections. The target for *Ng* DNA was a fragment of 102 bp of the porA pseudogene [30] and the target for *Tv* DNA was a fragment of 67 bp of a repeated sequence [31]. Two μL of sample DNA was added to 23 μL in the reaction solution for amplification with the following thermal cycling conditions: 50 °C for 2 min, 95 °C for 10 min, followed by 40 cycles at 95 °C for 15 s and at 60 °C for 1 min. Every PCR reaction included positive (*Ng* DNA and *Tv* DNA positive urine samples) and negative (water) controls.

### 4.4. Cytological Diagnosis

All HR-HPV and/or LR-HPV positive women were referred to cytology with the aim of implementing and motivating a counseling and prevention strategy for the control of STIs.

Pap smears collected from these women were evaluated according to the 2001 Bethesda System [32] at the LILT Healthcare Centre [26].

### 4.5. Statistical Analysis

Data were expressed as a median (IQR) and percentages (95% CI) as appropriate. Comparisons between groups were performed using the chi-square test or Mid-p exact test. A *p*-value < 0.05 was considered statistically significant (two-tail test). All statistical analyses were performed with OpenEpi software, version 3.01 [33].

## 5. Conclusions

This study emphasizes the importance of developing and implementing public health policies to improve the health of migrant women, focusing on the respect and the rights of migrants regardless of their immigration status. To this regard, more appropriate health services that adequately address all aspects of women's health, especially sexual and reproductive health issues, are required.

To our knowledge, this is the first Italian study focused on STI screening among undocumented migrant women. The high participation rate underlines the importance of providing urine-based STI screening at associations and/or organizations dealing with migrants. 

## Figures and Tables

**Table 1:** Socio-demographic and sexual health characteristics of 537 women enrolled: overall and by geographical World Health Organization (WHO) Region.

Characteristics of Enrolled Women	Overall		Latin America		Eastern Europe		Eastern Mediterranean		Western Pacific		Africa		Southeast Asia	
	*n*	%	*n*	%	*n*	%	*n*	%	*n*	%	*n*	%	*n*	%
	537	100	245	45.6	165	30.7	43	8.0	36	6.7	34	6.3	14	2.6
**Socio-demographic characteristics**														
Migration time *														
≤1 year	121	22.5	61	24.9	29	17.6	9	20.9	10	27.8	9	26.5	3	21.4
2–3 years	112	20.9	55	22.4	27	16.4	10	23.3	10	27.8	7	20.6	3	21.4
4–5 years	55	10.2	23	9.4	18	10.9	7	16.3	3	8.3	3	8.8	1	7.2
>5 years	249	46.4	106	43.3	91	55.1	17	39.5	13	36.1	15	44.1	7	50.0
**Marital status**														
Single or widow	134	24.9	63	25.7	36	21.8	14	32.5	5	13.9	16	47.1	0	0.0
Married	308	57.4	138	56.3	95	57.6	18	41.9	27	75.0	17	50.0	13	92.9
Divorced or separated	95	17.7	44	18.0	34	20.6	11	25.6	4	11.1	1	2.9	1	7.1
**Educational background**														
No qualification	29	5.4	2	0.8	20	12.1	5	11.6	0	0.0	3	8.8	0	0.0
Elementary school	83	15.4	31	12.7	24	14.6	11	25.5	5	13.9	6	17.6	5	35.7
Secondary school	47	8.8	24	9.8	14	8.5	6	14.0	1	2.8	1	3.0	1	7.2
High school	307	57.2	154	62.8	70	42.4	15	34.9	18	50.0	18	53.0	8	57.1
Degree	71	13.2	34	13.9	37	22.4	6	14.0	12	33.3	6	17.6	0	0.0
**Sexual and reproductive health characteristics**														
**Age at first intercourse**														
Virgins	7	1.3	0	0.0	3	1.8	3	7.0	0	0.0	1	3.0	0	0.0
≤15 years	94	17.5	47	19.2	28	17.0	6	14.0	1	2.8	6	17.6	6	42.9
16–19 years	238	44.3	117	47.8	83	50.3	13	30.2	14	38.9	10	29.4	1	7.1
>19 years	190	35.4	78	31.8	49	29.7	18	41.8	21	58.3	17	50.0	7	50.0
Don’t remember	8	1.5	3	1.2	2	1.2	3	7.0	0	0.0	0	0.0	0	0.0
**Sexual activity**														
No partner	138	25.7	65	26.5	41	24.8	10	23.2	6	16.7	13	38.2	3	21.5
Stable partner (>1 year) **	350	65.2	156	63.7	111	67.3	27	62.8	30	83.3	17	50.0	9	64.3
Recently partner (<1 year)	47	8.7	24	9.8	12	7.3	6	14.0	0	0.0	4	11.8	1	7.1
More partners	2	0.4	0	0.0	1	0.6	0	0.0	0	0.0	0	0.0	1	7.1
**Contraceptive methods**														
None	348	64.8	143	58.3	119	72.1	27	62.8	27	75.0	20	58.8	12	85.8
Condom	45	8.4	24	9.8	11	6.7	0	0.0	2	5.6	7	20.6	1	7.1
Pill	107	19.9	58	23.7	21	12.7	14	32.6	6	16.7	7	20.6	1	7.1
Others	37	6.9	20	8.2	14	8.5	2	4.6	1	2.7	0	0.0	0	0.0
**Pregnancies**														
None	161	30.0	78	31.8	38	23.0	20	46.5	4	11.1	18	52.9	3	21.5
1	140	26.1	61	24.9	44	26.7	10	23.2	13	36.1	7	20.6	5	35.7
2	121	22.5	48	19.6	48	29.1	9	20.9	12	33.3	2	5.9	2	14.2
3	70	13.0	33	13.5	23	13.9	2	4.7	5	13.9	3	8.8	4	28.6
>3	45	8.4	25	10.2	12	7.3	2	4.7	2	5.6	4	11.8	0	0.0
**Past Sexually Transmitted Diseases (STD symptomatic cases)**														
Yes	142	26.4	74	30.2	48	29.1	9	20.9	2	5.6	9	26.5	0	0.0
**Type of STDs**														
Condyloma	5	0.9	3	1.2	2	1.2	0	0.0	0	0.0	0	0.0	0	0.0
Syphilis	1	0.2	1	0.4	0	0.0	0	0.0	0	0.0	0	0.0	0	0.0
Gonorrhoea	1	0.2	1	0.4	0	0.0	0	0.0	0	0.0	0	0.0	0	0.0
Chlamydia	3	0.6	1	0.4	2	1.2	0	0.0	0	0.0	0	0.0	0	0.0
Genital Herpes	4	0.7	3	1.2	1	0.6	0	0.0	0	0.0	0	0.0	0	0.0
Bacterial vaginosis	37	6.9	20	8.2	10	6.1	1	2.3	1	2.8	5	14.8	0	0.0
Mycosis	36	6.7	17	6.9	14	8.5	4	9.3	0	0.0	1	2.9	0	0.0
Trichomoniasis	1	0.2	0	0.0	0	0.0	1	2.3	0	0.0	0	0.0	0	0.0
Bacterial vaginosis/mycosis	21	3.9	10	4.2	8	4.8	2	4.7	0	0.0	1	2.9	0	0.0
Others	33	6.1	18	7.3	11	6.7	1	2.3	1	2.8	2	5.9	0	0.0

* Migration time: time of residence in Italy; ** Stable partner (>1 year): same sex partner for at least one year. Sexually transmitted infection (STI).

**Table 2:** Age-stratified Human papillomavirus (HPV) prevalence of 537 women enrolled: overall and by geographical WHO Region.

Age-Stratified HPV Prevalence	Overall		Latin America		Eastern Europe		Eastern Mediterranean		Western Pacific		Africa		Southeast Asia	
	*n*	%	*n*	%	*n*	%	*n*	%	*n*	%	*n*	%	*n*	%
**<25 years**	27/83	32.5	17/42	40.5	9/32	28.1	1/7	14.3	0/0	0	0/2	0	0/0	0
**25–34 years**	35/161	21.7	20/75	26.7	7/48	14.6	3/15	20	2/4	50	3/15	20	0/4	0
**35–44 years**	34/131	26	16/60	26.7	5/32	15.6	2/12	16.7	5/12	41.7	3/11	27.3	3/4	75
**45–54 years**	22/103	21.3	11/44	25	6/32	18.7	2/8	25	3/13	23.1	0/2	0	0/4	0
**>55 years**	12/59	20.3	8/24	33.5	3/21	14.3	1/1	100	0/7	0	0/4	0	0/2	0
**TOT**	130/537	24.2	72/245	29.4	30/165	18.2	9/43	21	10/36	27.8	6/34	17.6	3/14	21.4

**Table 3:** Age-stratified *C. trachomatis* prevalence of 537 women enrolled: overall and by geographical WHO Region.

Age-Stratified *Ct* Prevalence	Overall		Latin America		Eastern Europe		Eastern Mediterranean		Western Pacific		Africa		Southeast Asia	
	*n*	%	*n*	%	*n*	%	*n*	%	*n*	%	*n*	%	*n*	%
**<25 years**	11/83	13.2	10/42	23.8	0/32	0	1/7	14.3	0/0	0	0/2	0	0/0	0
**25–34 years**	18/161	11.2	8/75	10.7	2/48	4.2	5/15	33.4	1/4	25	2/15	13.4	0/4	0
**35–44 years**	6/131	4.6	2/60	3.3	2/32	6.2	1/12	8.3	0/12	0	1/11	9.1	0/4	0
**45–54 years**	4/103	3.9	3/44	6.8	0/32	0	0/8	0	1/13	7.7	0/2	0	0/4	0
**>55 years**	3/59	5	1/24	4.2	2/21	9.5	0/1	0	0/7	0	0/4	0	0/2	0
**TOT**	42/537	7.8	24/245	9.8	6/165	3.6	7/43	16.3	2/36	5.6	3/34	8.8	0/14	0
